# Higher Charges for Distant Patients

**Published:** 1921-09-10

**Authors:** Hedley Lucas

**Affiliations:** House Governor and Secretary. The Queen's Hospital, Birmingham


### THE EDITOR'S LETTER-BOX.

**Correspondence on all subjects is invited, but we cannot in any way be responsible for the opinions expressed.**

### "Higher Charges for Distant Patients."

*To the Editor of* The Hospital.

Sir,—I regret that you "are still not clear why the Queen's Hospital, Birmingham, scheme applies only to patients living at a distance," but I think that the following extract from my previous letter indicates the reason :

"The scheme has been inaugurated because it is considered that patients (*i.e.*, those from a distance) who receive the special treatment which is afforded by the 'Queen's,' which is not only a general hospital but a medical school, and serves a wide area (from not a few districts of which little, if any, pecuniary support is forthcoming, and in which, be it noted, voluntary hospitals are situated), should financially assist the Queen's Hospital, especially as it has a large deficit."

"Distant" patients are considered as being in a different position from those in Birmingham, in that the former derive advantages of being admitted to the Queen's Hospital instead of to a local institution. Moreover, Birmingham patients assist the "Queen's" financially by contributing systematically to that city's various hospital funds, whereas those from outside areas (in which are to be found voluntary hospitals) rarely do.

I repeat that the object of my Committee is not "to encourage patients living at a distance to seek treatment at hospitals nearer than the Queen's to their own homes," nor has the scheme had any such consequence—its elasticity and the Queen's Hospital's reputation are sufficient safeguards. Yours faithfully,

Hedley Lucas,
House Governor and Secretary.

The Queen's Hospital, Birmingham,
September 3, 1921.

